# Nondestructive Detection and Quantification of Blueberry Bruising using Near-infrared (NIR) Hyperspectral Reflectance Imaging

**DOI:** 10.1038/srep35679

**Published:** 2016-10-21

**Authors:** Yu Jiang, Changying Li, Fumiomi Takeda

**Affiliations:** 1 College of Engineering, University of Georgia, Athens, Georgia, 30602, United States of America; 2 Appalachian Fruit Research Station, United States Department of Agriculture-Agricultural Research Service, Kearneysville, West Virginia, 25430, United States of America

## Abstract

Currently, blueberry bruising is evaluated by either human visual/tactile inspection or firmness measurement instruments. These methods are destructive, time-consuming, and subjective. The goal of this paper was to develop a non-destructive approach for blueberry bruising detection and quantification. Experiments were conducted on 300 samples of southern highbush blueberry (Camellia, Rebel, and Star) and on 1500 samples of northern highbush blueberry (Bluecrop, Jersey, and Liberty) for hyperspectral imaging analysis, firmness measurement, and human evaluation. An algorithm was developed to automatically calculate a bruise ratio index (ratio of bruised to whole fruit area) for bruise quantification. The spectra of bruised and healthy tissues were statistically separated and the separation was independent of cultivars. Support vector machine (SVM) classification of the spectra from the regions of interest (ROIs) achieved over 94%, 92%, and 96% accuracy on the training set, independent testing set, and combined set, respectively. The statistical results showed that the bruise ratio index was equivalent to the measured firmness but better than the predicted firmness in regard to effectiveness of bruise quantification, and the bruise ratio index had a strong correlation with human assessment (R2 = 0.78 − 0.83). Therefore, the proposed approach and the bruise ratio index are effective to non-destructively detect and quantify blueberry bruising.

The United States (U.S.) produced 239,000 tonnes of blueberries in 2013, accounting for 57% of total world production^1^. More than half of U.S. production went to the fresh fruit market and created over 579 million dollars in revenue^2^. Much of the blueberry crop destined for fresh market is still hand-harvested. Mechanical harvesting has a major limitation in that it creates more bruises which decrease fruit quality and ultimately reduce the monetary value of the blueberry crop^3^. In fact, fruit bruising causes around 10% of total economic losses of the blueberry industry every year. Additionally, bruising may accelerate other biological processes such as spoilage^4^. Even worse, rotten or fermented fruit could affect other healthy fruit, resulting in significant losses during long-distance transportation^5^.

Bruising is a type of subcutaneous tissue damage without rupturing fruit skin^6^. Typically, the tissue damage causes the mixture of phenolic compounds and polyphenol oxidase, generating dark coloration. Bruises are indicated by the discoloration of damaged tissues and thus they can be observed and differentiated from healthy tissues^6^. Consequently, visual inspection is an intuitive way to detect and assess fruit bruising. Bruises are not externally observable for most fruits, especially fruits with dark coloration such as blueberries. Therefore, visual inspection requires slicing fruit samples. Currently, each blueberry sample is sliced along the equatorial axis and the slices are imaged by color cameras. Human graders evaluate the color images and calculate the bruising level, the ratio of the area of the discolored region to the total surface area of the slice. Typically, a blueberry with a bruising level higher than 0.2 (20%) is considered bruised, and the percent of bruised fruit in each treatment is the bruise severity for that group. A limitation of the method is that it is impossible to detect bruises that are not present on the sliced cross-section. Moreover, illumination conditions could significantly affect inspection results.

In addition to discoloration, bruised tissues are typically softer than healthy tissues^6^. A palpation method is often used in which a blueberry is held between one’s thumb and index fingers and the fruit is gently squeezed and rolled. However, this method is subjective and laborious. Therefore, a number of automated approaches have been developed to measure fruit firmness that can be used as an indirect index for bruise quantification and assessment. Those approaches include firmness/texture analysis, acoustic impulse-response measurement^7^, and resonance frequency-based method^8^. Although these methods provide objective and repeatable measurements of fruit firmness, they are destructive to fruit samples. To overcome this issue, a laser air-puff detector was invented to measure fruit firmness in a non-contact manner^9^, and was applied to blueberry firmness measurement^10^. A fruit sample is deformed by a puff of air and a laser displacement sensor is used to record the deformation to assess the fruit firmness. Although the technique does not directly contact samples, it could cause potential damage after repeated measurements because fruit deformation in each measurement could be accumulated and ultimately result in fruit damage.

In recent years, advanced imaging modalities have been explored as non-destructive approaches for bruise detection. X-ray imaging was applied to detect bruises using the difference in radiation attenuation coefficients between bruised and healthy tissues. Typically, bruised tissues have lower density and absorb less radiation than healthy tissues. The results showed that X-ray imaging could achieve over 90% accuracy in detecting bruises in apples^11^. Nonetheless, X-ray imaging techniques have potential safety issues and X-ray instruments are expensive for agricultural applications. As a non-ionizing method, magnetic resonance imaging (MRI) was also explored due to the difference in free water (water released by damaged cells) between bruised and healthy avocado tissues^12^. In early stages, more free water was released in the bruised areas, resulting in higher intensity in MRI images. The results showed that MRI detected bruises immediately after impact, but it was not practical for the food industry or large-scale food research because of the high cost of the instruments. In addition, a dynamic thermal imaging (TI) method, pulsed-phase thermography (PPT), has been studied because of the difference in thermal diffusivity between bruised and healthy fruit^13^. When heating or cooling fruit samples, the diffusivity difference caused different rates of temperature change which were used to differentiate bruised and healthy fruit. PPT is less costly than X-ray imaging and MRI without safety concerns. However, the relatively low signal-to-noise ratio (SNR) and topological effects could cause potential issues for quantitative data analysis such as bruise quantification. To date, none of the three imaging modalities has been used for blueberry bruise detection.

Hyperspectral Imaging (HSI) has been investigated for fruit bruise detection because both physical and chemical property changes affect the spectral profile^14^. Previous studies have demonstrated the feasibility of using HSI reflectance mode to detect bruises in various fruits. In particular, the spectral range from 900 to 1700 nm was appropriate to detect bruises in apples^13^,^15^,^16^, pears^17^, and jujube^18^. In this spectral range, all the studies identified bruised tissues with over 88% accuracy, as the spectra of bruised tissues were significantly different from those of healthy tissues. The spectral difference between the bruised and healthy tissues was probably due to tissue disruption including cell wall failure and the release of free water from cells. The free water absorbed more light and affected the reflectance spectra of bruised tissues in the range from 900 to 1700 nm, especially at several key wavelengths with high water absorption such as 970 nm, 1200 nm, and 1470 nm^19^. Other than directly using them to identify bruised tissues, the spectra of the fruit could be correlated to fruit firmness by regression models such as partial least squares regression (PLSR)^20^. Two consecutive studies were conducted to predict blueberry firmness using reflectance and transmittance modes ranging from 400 to 1000 nm^21^,^22^. Results showed that the predicted firmness could be used for qualitative analysis of blueberry bruising, but the root mean squared error of predictions (RMSEPs) were 20% of the mean value of the fruit firmness, which might be insufficient for quantitative assessment of blueberry bruising.

In addition to its efficacy for detecting fruit bruising, HSI is also suitable for the food industry because it is safer than X-ray imaging, more affordable than MRI, and better in image quality than thermal imaging. In particular, the HSI reflectance mode could be applied to fruit packing lines for online sorting after modifications^14^. There was no HSI study done for blueberry bruising so far, and most HSI studies primarily explored spectral differences and did not take full advantage of the technique. Therefore, the present study utilized both the spatial and spectral information for blueberry bruising quantification by combining image processing algorithms and chemometrics.

The overall goal of this study was to develop a non-destructive approach using near-infrared (NIR) hyperspectral imaging (950 to 1650 nm) to detect and quantify blueberry bruising. Specific objectives were to (i) classify healthy and bruised tissues using the spectra extracted from the regions of interest (ROIs) of the SHB (southern highbush blueberry) and NHB (northern highbush blueberry) fruit, (ii) quantify blueberry bruising using the bruise ratio index, and (iii) compare the effectiveness of bruise quantification using the bruise ratio index, measured and predicted firmness, and human assessment.

## Results and Discussion

### Grayscale images at representative wavelengths

**Figure 1:**
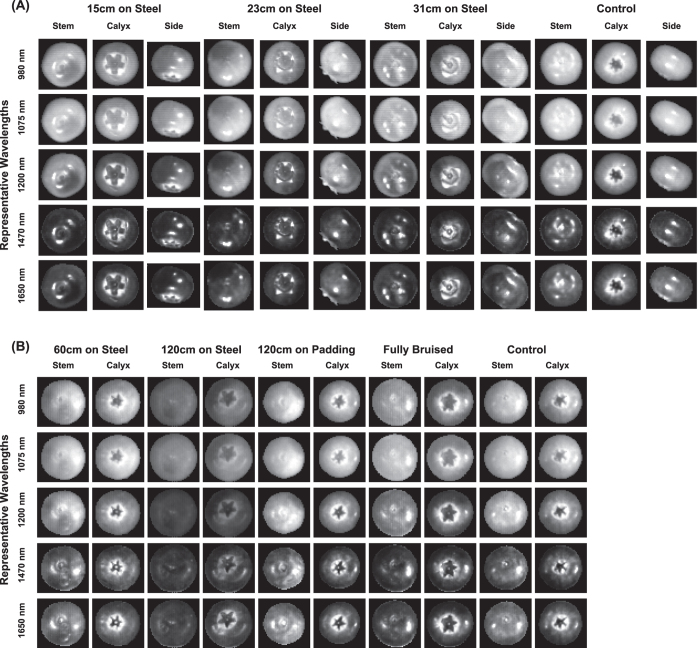
Grayscale images at representative wavelengths of blueberries: (**A**) for southern highbush cultivars (Dataset #1) and (**B**) for northern highbush cultivars (Dataset #2).

Grayscale images were observed at five representative wavelengths including three wavelengths for free water absorption (980, 1200, and 1470 nm) and two wavelengths for local peaks (1075 and 1650 nm). Generally, the blueberry samples of both southern and northern cultivars were hypo-intense (less bright) at higher wavelengths (1200, 1470, and 1650 nm) compared to lower wavelengths (980 and 1075 nm), whereas the background always absorbed most of the light at all five wavelengths (Fig. 1). In particular, the contrast between the samples and background was the highest at 1075 nm because the samples absorbed the least light at this wavelength. Therefore, the grayscale images at 1075 nm can be used to segment the blueberry samples from the background.

On each sample, healthy tissues were hyper-intense (brighter) while bruised tissues were hypo-intense (less bright) at 980, 1075, and 1200 nm. Both healthy and bruised tissues were hypo-intense at 1470 and 1650 nm. The contrast between the healthy tissues and bruised tissues was the highest at 1200 nm. Therefore, grayscale images at 1200 nm were used to manually select ROIs of healthy and bruised tissues for spectra extraction.

One exception was the calyx end, which appeared as dark as bruised tissues in most wavelengths. However, this phenomenon was observed from all the samples in both control and bruising treatments, and thus the low reflectance intensity was not due to bruises. In fact, compared with other parts of the fruit, the calyx end typically is harder to bruise so the low reflectance intensity was most likely because the calyx end might have special chemical or physical properties, leading to more light absorption and less reflection. As a result, to accurately detect and quantify bruises, it was necessary to exclude the calyx end of each fruit in the following hyperspectral image classification and bruise ratio index calculation.

### Reflectance spectra of healthy and bruised tissues

**Figure 2:**
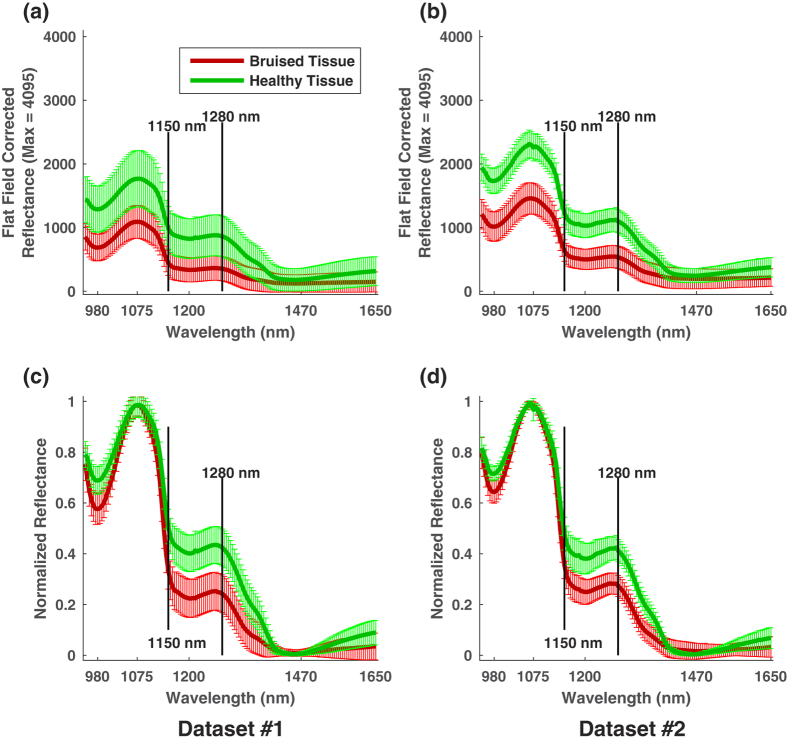
Mean spectra (solid line) and standard deviation (error bar) of healthy and bruised tissue. (**a**,**b**) Are in flat field corrected reflectance for southern and northern cultivars, respectively; and (**c**,**d**) are in normalized reflectance for southern and northern cultivars, respectively.

Since the present study focused on bruise detection and quantification, experimental variables were not discussed if they did not affect the separation between healthy and bruised tissues. According to statistical test results, the closest mean spectra of healthy and bruised tissues were statistically separable regardless of development time (see Supplementary Fig. S1, Fig. S2, and Table S1) and blueberry cultivar (see Supplementary Figs S3–S6, Table S2, and Table S3). Therefore, the spectral difference between healthy and bruised tissues was not affected by the two variables (Fig. 2). Prior to spectral normalization, there was no obvious shape difference in the mean spectra of healthy (or bruised) tissues between southern (Dataset1) and northern (Dataset2) highbush blueberry cultivars (Fig. 2(a,b)). Although the intensity of the spectra from NHB cultivars was on average higher than that of SHB cultivars, the difference was primarily caused by different illumination conditions. After removing this effect by spectral normalization, the mean spectra showed no obvious difference in both shape and intensity between SHB and NHB cultivars (Fig. 2(c,d)). Therefore, the spectra extracted from one experiment can be used to train a classifier to differentiate the spectra extracted from another experiment.

The mean spectra between healthy and bruised tissues in both SHB and NHB cultivars were clearly different (Fig. 2). Regarding the spectral shape, the mean spectra of healthy and bruised tissues can be grouped into three segments, i.e., 950 to 1150 nm, 1150 to 1280 nm, and 1280 to 1650 nm. The spectra of healthy and bruised tissues showed little or no difference in the first two segments but a significant difference in the third one. In the third spectral range from 1280 to 1650 nm, the intensity of the spectra decreased to the minimum at 1470 nm, and then began to increase for healthy tissues while being flat for bruised tissues between 1470 and 1650 nm. After spectral normalization, the upward trend of the spectral intensity of healthy tissues became more obvious (Fig. 2(c,d)), which could be useful to classify healthy and bruised tissues.

In addition, two local minima (at 980 and 1200 nm) and the global minimum (at 1470 nm) matched typical absorption wavelengths of liquid water^19^, confirming the assumption that fruit bruising causes redistribution of free water in fruit tissues. According to a previous study^23^, bruises cause damage to cell membranes and result in leaking free water. The free water stays around the bruised tissues in early stages, and then diffuses away and evaporates out of the fruit, resulting in dry cavities in late stages. Therefore, in early stages, bruised tissues contain more free water than healthy tissues and thus absorb more light at certain wavelengths with high liquid water absorption.

In terms of the spectral intensity, prior to spectral normalization, the intensity of the mean spectra of healthy tissues were always higher than that of bruised tissues (Fig. 2(a,b)). The spectra of healthy and bruised tissues in southern cultivars (Dataset1) had a larger overlap than the spectra in northern cultivars (Dataset2). This occurred mainly because of the differences in manual ROI selection between the two experiments. Compared with Dataset2, Dataset1 had no fully-bruised treatment dedicated for spectra extraction of bruised tissues. Therefore, the selected ROIs for bruised tissues in Dataset1 might have higher possibility of mixing with healthy tissues than those in Dataset2, ultimately resulting in a large spectral overlap between two treatments. After spectral normalization, the differences between healthy and bruised tissues were most prominent at three ranges: 950 to 1000 nm, 1150 to 1400 nm, and 1500 to 1650 nm. Potentially, wavelengths in these three ranges could be used for feature selection and improvement of the classification performance.

### Classification results

#### Classification of spectra extracted from ROIs

Both the 10-fold cross-validation on the training set and the evaluation on independent test sets achieved accuracies of over 92%, indicating the proposed detecting method was accurate and robust (Table 1). The accuracies of the 10-fold cross-validation were over 94% with a variation up to 0.34% because normalized spectra of healthy and bruised tissues within each dataset were clearly separated with little overlap. Accuracies decreased 2.35% and 3.79% when using SpectraSet2 and SpectraSet1 as independent test set, respectively. The performance reduction was largely because testing the classifier using an independent test set is more rigorous than k-fold cross-validation. It should be noted that since all the parameters used in the SVM classifier were set to default values, they might not be optimal for all cases, and thus parameter optimization could further improve the accuracy. In addition, information at some wavelengths might be redundant features for classification, so feature selection could improve the classification performance as well.

Overall, the 10-fold cross-validation on the combined spectral data from SpectraSet1 and SpectraSet2 achieved over 96% accuracy for the test sets (Table 2). Although the accuracy of classifying pixels of healthy tissues (true negative) was slightly higher (2.26%) than that of bruised tissues (true positive), they were comparable classification results with satisfactory accuracies. In addition, the classification performance on the combined spectral library was stable (performance variation was up to 0.21%). It should be noted that these performances are based on the spectral data extracted from the selected ROIs and the performance on all the images at the pixel level may vary.

#### Image classification

**Figure 3:**
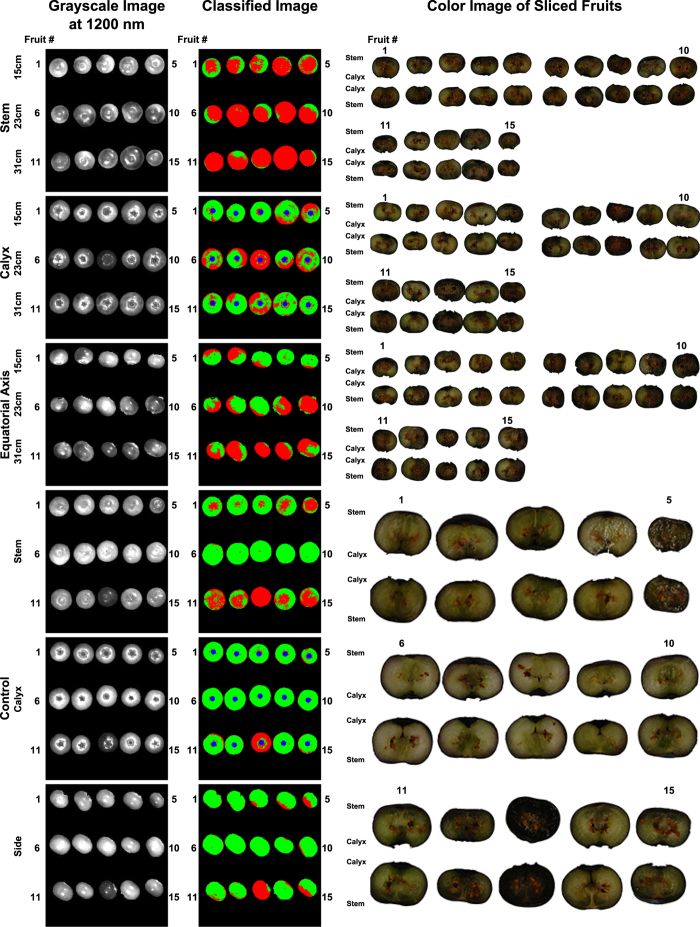
Grayscale image at 1200 nm, classified image, and color image of sliced fruit of representative results from Dataset1 (southern highbush cultivars). Red and green color represent bruised and healthy tissues, respectively.

For Dataset1, the proposed method recognized bruises using hyperspectral images, and the results closely matched the observations of bruises in the color images of the sliced fruit (Fig. 3). The blueberry samples were sliced perpendicular to the bruise position, which was controlled in the experiment, so the bruises were easily observed on the slicing plane. In addition, it was noteworthy that the pendulum wooden arm with the sample holder (22.5–29 g) was 15 times heavier than a typical blueberry sample (1.5–2 g), and thus bruises created by the pendulum were significantly more severe than those created by randomly dropping the fruit onto a steel surface from the same height. Therefore, the bruises created in the first experiment were clearly discolored compared to healthy tissues. Some samples in control groups were highly or fully bruised in both image classification results and color images of sliced fruit, because they may have been damaged during transportation prior to the experiments. The bruises were more severe on the stem side and equatorial axis than on the calyx side, which validated the assumption that calyx end was harder to bruise.

**Figure 4:**
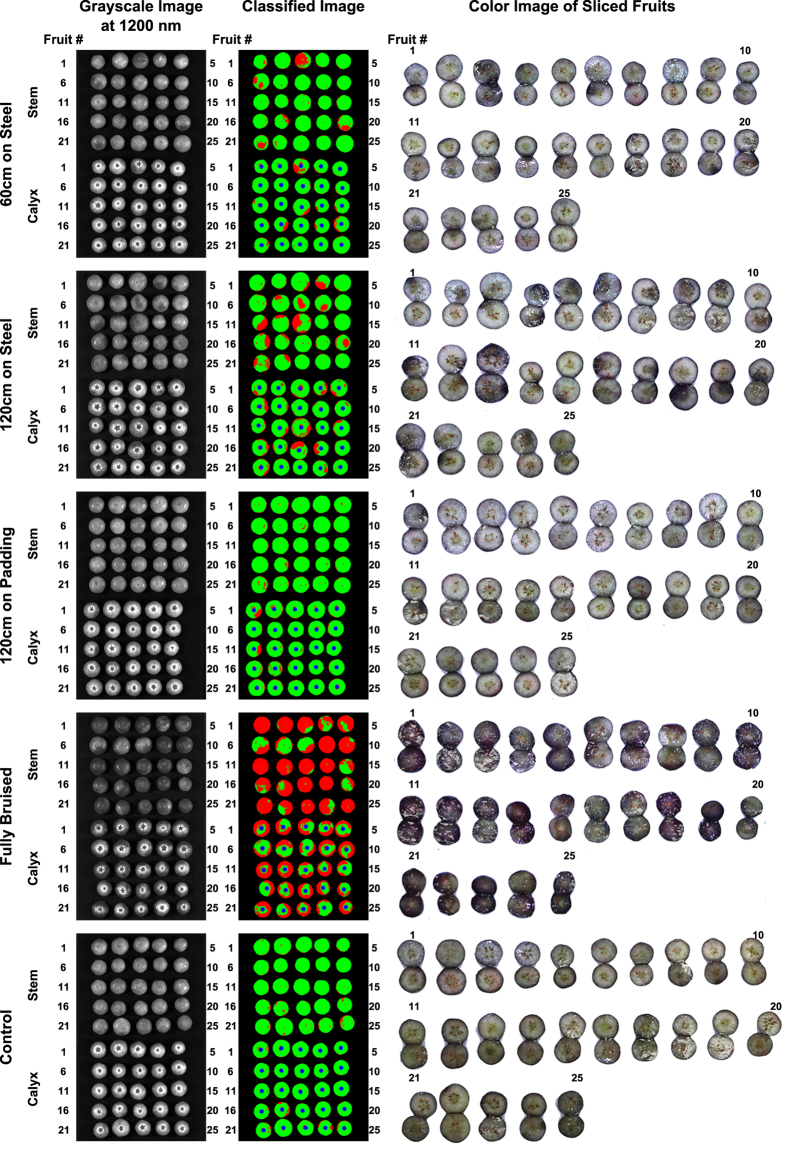
Grayscale image at 1200 nm, classified image, and color image of sliced fruit of representative results from Dataset2 (northern highbush cultivars). Red and green color represent bruised and healthy tissues, respectively.

For Dataset2, although the proposed method identified bruises, sometimes the results did not match with the observations of bruises in the color images of the sliced fruit (Fig. 4). This occurred mainly because different bruise creation and slicing methods were used in the second experiment. Compared with those created by the pendulum, the bruises created by randomly dropping from certain heights were less severe and tended to be shallower. Consequently, if blueberry samples were only sliced along the equatorial axis, the bruises would not be observed on the slicing plane when they occurred at shallow positions not along the equatorial axis. The bruises were consistently more severe on the stem side than on the calyx side. In addition, the amount of bruises and their severity were related to the drop height and the impact surface^24^. The classified images clearly showed this pattern. When samples were dropped from different heights onto the same steel surface, more bruises (red areas) were observed in the samples with higher drop heights. When samples were dropped onto different surfaces from the same height, less bruises (red areas) were observed in the samples dropped onto the padded surface than onto the steel surface.

### Comparison between bruise ratio index and traditional measurements

**Figure 5:**
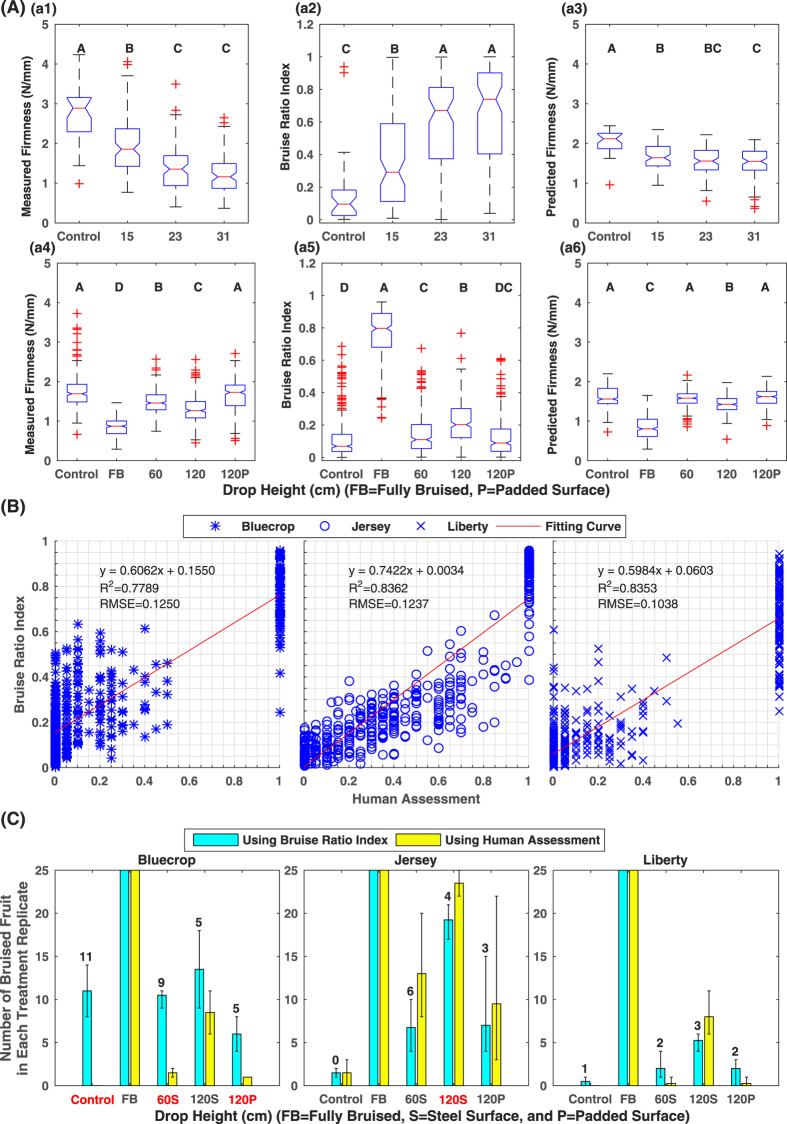
Statistical analysis results in the present study. Panel (A): boxplot of measured firmness, bruise ratio index extracted from HSI, and predicted firmness by PLSR. Treatments with different letters are statistically significant with each other (see Supplementary Tables S4–S9), and treatment mean values of each index are sorted alphabetically. (a1–a3) and (a4–a6) are measured firmness, bruise ratio index, and predicted firmness for Dataset1 (southern cultivars) and Dataset2 (northern cultivars), respectively. Panel (B): linear regression between bruise ratio index and human assessment for three northern cultivars including Bluecrop, Jersey, and Liberty. Panel (C): Comparison between the number of bruised fruit calculated by bruise ratio index and human assessment using a threshold value of 0.2. The red color of the treatment name indicated the results calculated by the bruise ratio index were statistically different from that calculated by human assessment (see Supplementary Tables S10–S12).

The statistical patterns obtained using bruise ratio index matched the patterns calculated using firmness measurement (Fig. 5(Aa1). When the drop height was increased, more bruises were created, and thus higher bruise ratio index and lower firmness of blueberry samples were measured. In addition, the results showed the same statistical significance among the treatments when using bruise ratio index and measured firmness, respectively (see Supplementary Tables S4–S7). Therefore, the bruise ratio index calculated by the proposed non-destructive method was equivalent to measured firmness in the statistical tests.

Although it showed a certain efficacy, firmness predicted by the PLSR model did not show statistical differences for some treatments (Fig. 5(Aa1). In Dataset1, the predicted firmness of the control treatment was statistically higher than that of the bruise treatments. However, the predicted firmness of bruise treatments dropped from 15 cm was not statistically higher than that of the treatments dropped from 23 and 31 cm, and thus the predicted firmness could not accurately represent bruises caused by different drop heights (see Supplementary Table S8). In Dataset2, although the treatments dropped onto steel and padded surfaces were statistically different, there was no significant difference between the control and bruise treatments dropped from 60 cm onto a steel surface and from 120 cm onto a padded surface (see Supplementary Table S9). Thus, the predicted firmness had the effectiveness of bruising assessment in certain situations, but it could not quantify bruising as accurately as the bruise ratio index. These observations agreed with previous studies^21^,^22^ which showed that the predicted firmness had a relatively large RMSEP using either reflectance or transmittance spectra. Therefore, compared with the predicted firmness, bruise ratio index could be a more effective index to non-destructively quantify and assess blueberry bruising.

**Figure 6:**
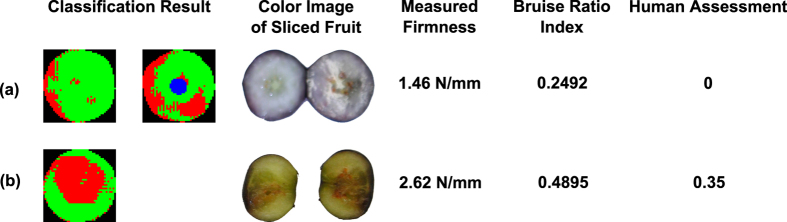
Inconsistent cases between bruise ratio index and traditional indices: (**a**) in consistency between bruise ratio index and human assessment (**b**) inconsistency between bruise ratio index and measured firmness.

Overall, the R^2^ value (0.78–0.84) indicated that bruise ratio index was strongly correlated with human assessment for northern cultivars (Fig. 5(B)). However, the Root Mean Squared Error (RMSE) was up to 12.5% of the bruise ratio index range (0 to 1), suggesting that bruise ratio index might be considerably different from human assessment in some treatments. In fact, the correlation analyses were further validated by the results of the thresholding classification of bruised fruit (Fig. 5(C)). For most cases, the differences between the number of bruised fruit calculated using bruise ratio index and human assessment were within 5 and they were not statistically different (see Supplementary Tables S10–S12). This confirmed that bruise ratio index was correlated with human assessment. However, for some cases such as the control group of Bluecrop, the number of bruised fruit calculated using bruise ratio index was significantly different from that calculated using human assessment (treatment names in red in Fig. 5(C), see Supplementary Tables S10–S12). Although human visual inspection was the most intuitive approach to evaluate and quantify blueberry bruising, it can only observe and evaluate bruises that developed on the slicing plane. For those bruises that did not occur on the equatorial axis or the slicing plane, human graders could not observe and evaluate them. For instance, a sample with a firmness of 1.46 N/mm was graded as healthy fruit with no bruised tissue, whereas the proposed imaging method measured a bruise ratio index of 0.2492 (Fig. 6(a)). To further explain this inconsistency between the human assessment and bruise ratio index, more advanced imaging techniques such as MRI need to be explored to characterize blueberry bruising.

Additionally, inconsistencies between measured firmness, bruise ratio index, and human assessment were observed. For instance, a blueberry with a firmness of 2.62 N/mm had a bruise ratio index of 0.4895 and a human assessment of 0.35 (Fig. 6(b)). Both bruise ratio index and human assessment were high, indicating that the fruit had bruises on it. However, the firmness measurement was high as well, contrary to the fact that bruised berries tend to be softer. This likely occurred because the instrument only measures the firmness in a local area. If a bruise develops 90 degrees from the measuring axis of the instrument, the fruit firmness reading may be greater than that measured along the bruising axis. Nevertheless, the fact was that this sample had spectra similar to bruised fruit but a firmness value similar to healthy fruit. This could explain the reason that the predicted firmness had large RMSEP, which was not good for accurately quantifying blueberry bruising. If a training set contains samples like the fruit in Fig. 6(b), the training process will force the PLSR model to fit those data, which may lead to large errors in predicting the firmness of common samples.

Bruise ratio as an index calculated by a non-destructive imaging approach showed a strong correlation with human assessment measured in a destructive manner. In addition, in the statistical tests, bruise ratio index was equivalent to firmness measured by instruments but better than firmness predicted by using hyperspectral imaging with the PLSR model. Therefore, bruise ratio index could be an effective index for quantification and assessment of blueberry bruising. Potentially, this method could also be used for assessing bruises of other berry fruits.

## Conclusions

The proposed non-destructive approach based on NIR hyperspectral imaging was an accurate and stable method to detect blueberry bruising. Compared with traditional indices, bruise ratio index was an effective index for quantification of blueberry bruising. Therefore, the proposed method and index could be used to non-destructively quantify and assess blueberry bruising by both researchers and the commercial industry. Future studies will be focused on feature selection and classifier optimization to further improve the efficiency and accuracy of the method, enabling the proposed method for online sorting of bruised blueberries.

## Materials and Methods

### Sample collection and preparation

Two experiments were conducted in this research (Fig. 1). The first experiment was conducted to study the spectral differences between healthy and bruised tissues in southern highbush cultivars, and bruises were manually created at controlled positions (see Supplementary Table S13). A total of 300 blueberry samples of three southern cultivars including Camellia, Rebel, and Star were collected in May 2015 from a commercial farm in Alma, Georgia, USA. Each cultivar contained 100 samples that were divided into four groups: a control treatment of 10 samples and three bruise treatments with 30 samples each. The control treatment was kept intact, whereas the three bruise treatments were dropped onto a steel surface from heights of 15, 23, and 31 cm, respectively. In order to control the position of fruit bruising, bruises were created by a specially-designed pendulum. The wooden arm of the pendulum was connected with a sample holder made of silicon rubber through a screw eye. For the screw eye, the thread end was installed into the wooden arm, and the loop end was adhered to the back face of the sample holder. The steel screw eye enables the sample holder to be attached and released by a switchable magnet, facilitating consistent sample dropping. For each blueberry sample, one side was stuck on the sample holder using petroleum jelly when the sample holder was attached by the magnet, and the other side would hit the contacting surface when the sample holder was released. Therefore bruises mainly occurred on the impacted face. After bruise creation, all the samples were divided equally into two groups and stored in an air-conditioned room (23 °C with 30–35% relative humidity) for 24 and 48 hours, respectively, before they were imaged.

The second experiment was conducted to study the spectral differences between healthy and bruised tissues for northern highbush cultivars, and the bruises were created by randomly dropping the fruit from certain heights (see Supplementary Table S13). A total of 1500 blueberry samples of three northern highbush cultivars including Bluecrop, Jersey, and Liberty were collected in August 2015 from a commercial farm in Grand Junction, Michigan, USA. Each cultivar contained 500 samples that were divided equally into five treatments, with each treatment sub-divided into four replicate groups of 25 samples each. The five treatments were: control; fully-bruised (dropped from 90 cm onto a steel surface 8 times); treatments dropped onto a steel surface from 60 and 120 cm; and onto a padded surface from 120 cm. The purpose of the fully-bruised treatment was to easily and accurately extract the spectra of bruised tissues because random dropping was not able to control the bruise position and could cause difficulties in spectral extraction and classifier training. After bruise creation, all samples were stored in an air-conditioned room (23 °C with 20–25% relative humidity) for 24 hours, and were then used for further image acquisition and processing.

### Hyperspectral image acquisition

**Figure 7:**
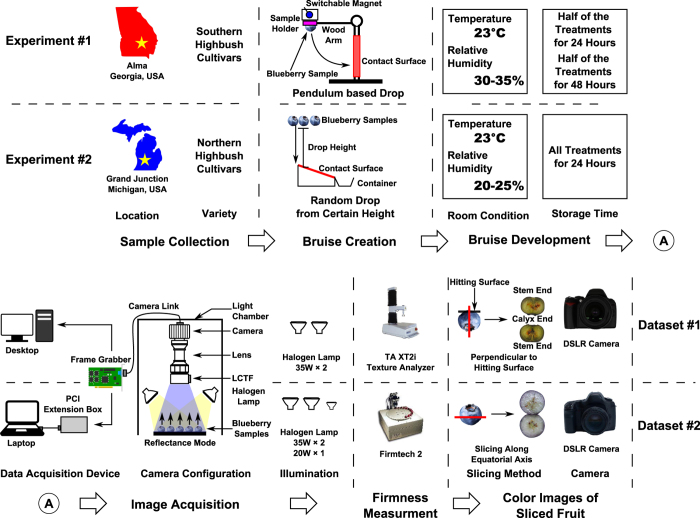
Overall flowchart of the two experiments conducted in this research.

The samples of the first experiment were imaged using a hyperspectral imaging system previously built by the Bio-Sensing and Instrumentation Laboratory of the University of Georgia^25^. All images were acquired in a light chamber to avoid the interference of ambient light (Fig. 7), and two 12 V 35 W halogen lamps (S4121, Satco Products Inc., NY, USA) were used as the illumination source. Prior to collecting images, an image of a 99% reflective panel (SRT-99-050, Labsphere Inc., North Sutton, NH, USA) was obtained as the white reference, and an image taken with the optical lens being covered was obtained as the dark reference. As the position of bruises was controlled, the samples were placed on a black cardboard holder with the bruised surfaces facing toward the camera. Control samples were imaged from three angles (stem, calyx, and equatorial axis). A total of 24 hyperspectral images were collected including 12 images for 150 samples stored for 24 hours and 12 images for 150 samples stored for 48 hours (see Supplementary Fig. S7 and Table S14 for detailed image layout).

The samples of the second experiment were imaged using a hyperspectral imaging system based on a portable light chamber and data acquisition device (Fig. 7). A frame grabber (PCI-1426, National Instruments Corp., TX, USA) was installed in a PCIe expansion box (NA 211A-NB, Netstor Technolog, Taiwan, China) through a PCIe to PCI adapter (ST369, Sintech Electronic, Shenzhen, China). The frame grabber was used to connect the camera of the hyperspectral imaging unit and a laptop. In addition to the two 12 V 35 W halogen lamps, a 12 V 20 W halogen lamp (PC 81763, GE Lighting, OH, USA) was used to enhance the uniformity of the illumination. White and dark reference images were collected using the same procedure as used in the first experiment. Since the position of bruising was not controlled in the second experiment, it was necessary to image both the stem and calyx sides. A total of 120 (5 treatments × 4 treatment replicates × 3 cultivars × 2 sampling positions) hyperspectral images were acquired, and each image contained 25 samples from a treatment replicate. All acquired hyperspectral images covered 141 wavelengths ranging from 950 to 1650 nm with a spectral interval of 5 nm.

### Reference measurements

#### Firmness

After image acquisition, the firmness of the blueberry samples was measured for reference. In the first experiment, a texture analyzer (TA. XT2i Texture Analyzer, Texture Technologies Corp., NY, USA) was used to measure firmness by following the procedure proposed by^22^. Each blueberry fruit was compressed between two parallel plates at a constant velocity of 1 mm/s for a total deformation of 3 mm. The firmness was calculated from the slope (N/mm) of the force/deformation curve between 0.5 mm and 2.5 mm displacement, as the curve was relatively straight in this range. In the second experiment, a FirmTech machine (FirmTech 2, BioWorks, Inc., KS, USA) was used to directly measure firmness (N/mm) of individual berry samples.

#### Color image of sliced fruit and human evaluation

After firmness measurement, the blueberry samples were sliced and imaged by a color camera for reference and human evaluation. In the first experiment, the samples were sliced perpendicular to the bruise position. For instance, if a bruise was created on the stem side of a fruit, the fruit was to be sliced along the stem-to-calyx axis, enabling an evaluator to observe the bruise on the slicing plane. The sliced samples were imaged by a digital single lens reflex (DSLR) camera (D40, Nikon Corp., Japan) under ambient illumination. As the slicing method was different from the approach used in a previous study^26^, these color images of the sliced samples were only used for reference. However, in the second experiment, the samples were sliced by following the protocol described by Brown^26^. The sliced samples were imaged by another DSLR camera (5D Mark II, Canon Inc., Japan) under ambient illumination. These color images were used for both reference and human evaluation. Trained human graders calculated the number of pixels of the discolored (bruised) area and the total cross-sectional area of each sample, and the ratio between the two was used as the human assessment of the bruising level of a sample^26^. In total, the two experiments produced two datasets (Dataset1 for experiment #1 and Dataset2 for experiment #2), and each dataset contained hyperspectral images, firmness measurements, and color images of sliced fruit (human assessment of bruising level only for Dataset2).

### Hyperspectral image processing

#### Automatic blueberry segmentation

**Figure 8:**
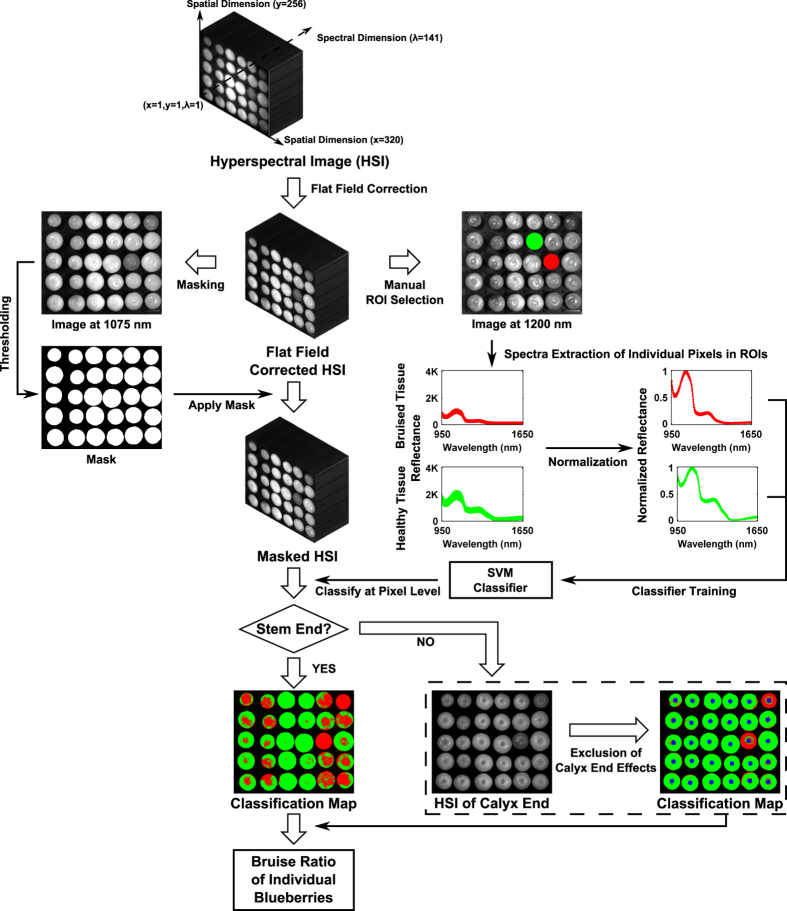
Flowchart of the hyperspectral image processing from flat field correction to calculation of the bruise ratio index.

The hyperspectral images were preprocessed by flat field correction to remove artifacts caused by non-uniform illumination or variations in the pixel-to-pixel sensitivity of the detector (Fig. 8). The process was executed in a customized program developed in IDL (IDL 7.1, Exelis Inc., VA, USA). Subsequently, the grayscale images at 1075 nm were used to create masks for segmenting individual samples from the background. The masks were automatically generated by thresholding. The grayscale images were thresholded at intensity levels of 737 and 942 (out of 4095) for Dataset1 and Dataset2, respectively. The resulting images were enhanced by morphological operations to fill holes and remove noise, and the refined results were used as masks. In addition, individual samples were able to be recognized by calculating connected components in the masks. The operations were implemented in MATLAB (MATLAB 2015b, The MathWorks Inc., MA, USA).

#### Manual ROI selection and spectral extraction

To accurately extract the spectra of healthy and bruised tissues, regions of interests (ROIs) were manually selected on the grayscale images at 1200 nm in ENVI (ENVI 4.7, Exelis Inc., VA, USA); this wavelength was chosen because the contrast between the bruised and healthy tissues was most prominent at this wavelength. For Dataset1, to accurately extract the spectra of healthy and bruised tissues, a total of 31 samples were used for ROI selection, including 14 fully-healthy and 17 fully-bruised samples. For Dataset2, a total of 600 (300 per treatment) samples from control and fully-bruised treatments were used for spectra extraction. One ROI was drawn on each selected sample, and the spectra of individual pixels in the ROIs were extracted. To balance the number of the extracted spectra, equal numbers of spectra for the two classes were selected from the extracted spectra. For the class with less spectra, all the spectra were kept, whereas for the class with more spectra, a subset of the spectra were randomly selected to keep the number of the spectra the same as the other class. Consequently, two spectral libraries (SpectraLib1 and SpectraLib2) were collected consisting of spectra of 28352 and 61580 pixels, respectively, and each contained equal numbers of spectra for healthy and bruised tissues.

#### Spectra and image classification

Support Vector Machine (SVM) was used for classification in this research. The SVM classifier was implemented by LibSVM (a software library for SVM classification)^27^, and the training and evaluation were executed in MATLAB. All classifier parameters were in default values as the objective of this research was to explore the classification performance baseline of the proposed method. Three methods were used to evaluate the efficacy of the hyperspectral imaging approach: (a) two classifiers were trained and evaluated on each individual spectral library by 10-fold cross validation, respectively; (b) the classifier was trained on one spectral library but evaluated on another library; and (c) the classifier was trained and evaluated on the combined spectral library by 10-fold cross-validation. It should be noted that all three methods were for classification of the spectra extracted from ROIs.

The classifier trained by the combined spectral library was used as the final model to classify the masked hyperspectral images at the pixel level. According to a previous study^24^, the spectra of the calyx end were unrelated to bruises but often misclassified as bruised tissues, and thus the calyx end needed to be excluded in image classification. The calyx end was a 5-pixel-radius circle located at the center of each connected component in the masks, and the pixels within the circle were excluded during classification.

Based on the image classification results, bruise ratios were calculated to quantify bruises on each fruit. Since the samples in Dataset1 were only imaged on one side, the bruise ratio index of each sample was calculated by the ratio of the number of pixels classified as bruised to the total number of fruit pixels. However, in the results of Dataset2 where each fruit was imaged on two sides, the bruise ratio index of each sample was calculated by averaging the ratio of each half.

#### Firmness prediction

Hyperspectral imaging has been used to predict fruit firmness by training regression models using the mean spectra of each fruit^14^. For comparison purposes, mean spectra of individual blueberry samples were extracted from the masked hyperspectral images, and the extracted spectra of each fruit with the measured firmness were used to train a PLSR model for fruit firmness prediction. According to previous studies^21^,^22^, to avoid effects caused by different initial conditions of blueberries, the spectra extracted from one experiment were not used to predict firmness of the samples in another experiment. Therefore, 300 and 1500 mean spectra were extracted and used for firmness prediction in the first and second experiments, respectively. The predicted firmness was used as an indirect index for blueberry bruising quantification and assessment.

### Statistical tests and comparison

To rigorously prove the potential of using the hyperspectral imaging system for bruise identification, multivariate analysis of variance (MANOVA) tests were conducted to compare the extracted spectra of healthy and bruised tissues of different cultivars. All 141 wavelengths were considered as variables, and they were multivariate-normally distributed.

To explore the effectiveness of bruise quantification and assessment, bruise ratio index was compared with measured firmness, firmness predicted by PLSR, and human assessment of bruising level. Since firmness is an indirect index for bruising quantification and assessment, bruise ratio index is not directly comparable with firmness values. Multiple comparisons followed by Kruskal-Wallis test (nonparametric equivalent to analysis of variance test) were conducted to compare the differences among various treatments when using the bruise ratio index, measured and predicted firmness, respectively, as the data were not normally distributed. Two indices were considered to be equivalent if they showed the same statistical pattern among various treatments.

Since human assessment is also an area ratio of bruised tissue to cross-section of a fruit sample, the bruise ratio index and human assessment are comparable. The correlation between human assessment and the bruise ratio index was analyzed by using linear regression and the coefficient of determination (R^2^). Additionally, a threshold was used to classify each fruit according to its bruise ratio index and human assessment, respectively. A fruit was classified as bruised if its bruise ratio index (or human assessment) exceeded a threshold of 0.2 (20% bruised area) in the present study. Analysis of variance (ANOVA) tests were conducted to compare the difference between the number of bruised fruit calculated using the bruise ratio index and human assessment for each treatment. The calculated number of bruised fruit in individual treatments were normally distributed.

The MANOVA and ANOVA tests were performed in SAS (package glm, SAS 9.3, SAS Institue Inc., NC, USA), whereas the multiple comparisons followed by Kruskal-Wallis test were conducted in R 3.2.4^28^ (package asbio). All tests were two-tailed and used a significance level of 0.05.

## Additional Information

**How to cite this article**: Jiang, Y. *et al*. Nondestructive Detection and Quantification of Blueberry Bruising using Near-infrared (NIR) Hyperspectral Reflectance Imaging. *Sci. Rep.* **6**, 35679; doi: 10.1038/srep35679 (2016).

## Supplementary Material

Supplementary Information

## Figures and Tables

**Table 1:** Classification performance of using independent test set.

Training set	Test set	10-fold cross validation on training set	Accuracy on test set
SpectraLib1	SpectraLib2	94.68 ± 0.34%	92.41%
SpectraLib2	SpectraLib1	97.05 ± 0.15%	93.29%

SpectraSet1 consisting of 28352 spectra extracted from Dataset1 (southern cultivars) and SpectraSet2 consisting of 61580 spectra extracted from Dataset2 (northern cultivars).

**Table 2:** Confusion matrix of 10-fold cross validation on the combined spectra from Dataset1 and Dataset2.

	Classified as bruised	Classified as healthy	Total
Actual as bruised	42773 (95.12%, true positive)	2193 (4.88%, false negative)	44966
Actual as healthy	1179 (2.62%, false positive)	43787 (97.38%, true negative)	44966
Overall Accuracy: 96.25 ± 0.21% for test			
