# Supplementary material for: Nondestructive Detection and Quantification of Blueberry Bruising using Near-infrared (NIR) Hyperspectral Reflectance Imaging
Source: Sci Rep. 2016 Oct 21;6:35679. doi: 10.1038/srep35679 (PMC5073368; doi:10.1038/srep35679)
Supplement: Supplementary Information [file srep35679-s1.pdf]

Nondestructive Detection and Quantification of Blueberry Bruising using Near-infrared (NIR) Hyperspectral Reflectance Imaging

Supplementary Materials

Yu Jiang[1], Changying Li[1,*], and Fumiomi Takeda[2]

[1]College of Engineering, University of Georgia, Athens, Georgia, 30605, United States of America

[2]Appalachian Fruit Research Station, United States Department of Agriculture-Agricultural Research Service, Kearneysville, West Virginia, 25430, United States of America

[*] Corresponding author at 712F Boyd Graduate Studies, 200 D. W. Brooks Drive, University of Georgia, Athens, Georgia, 30602, United States of America. Phone: (706) 542-4696; Fax: (706) 542-2475; Email: cyli@uga.edu; Website: http://sensinglab.engr.uga.edu/.

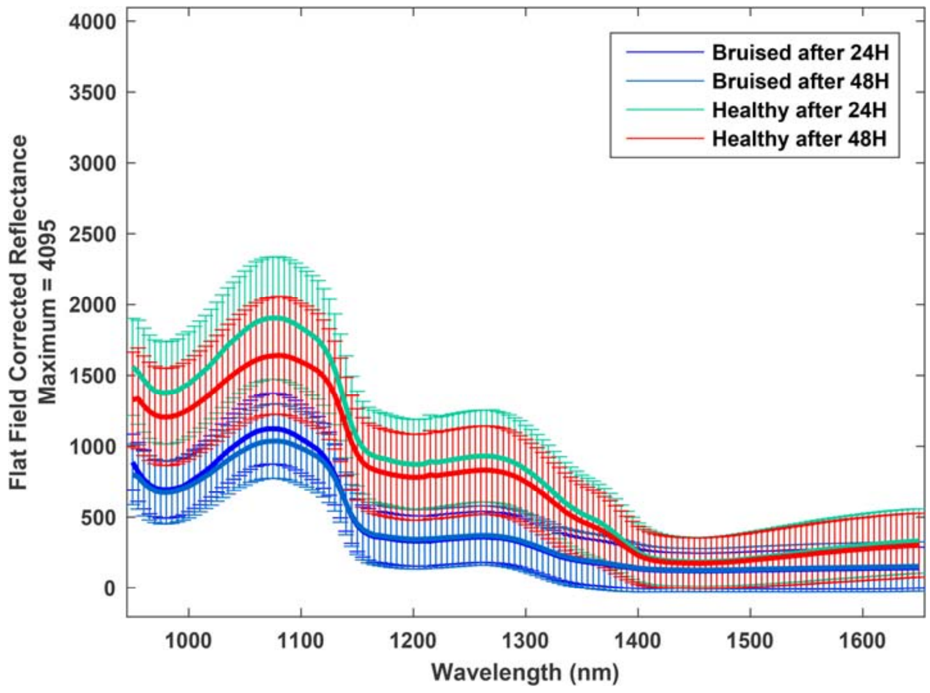

Figure S1. The spectra of healthy and bruised tissues stored for 24 and 48 hours, respectively.

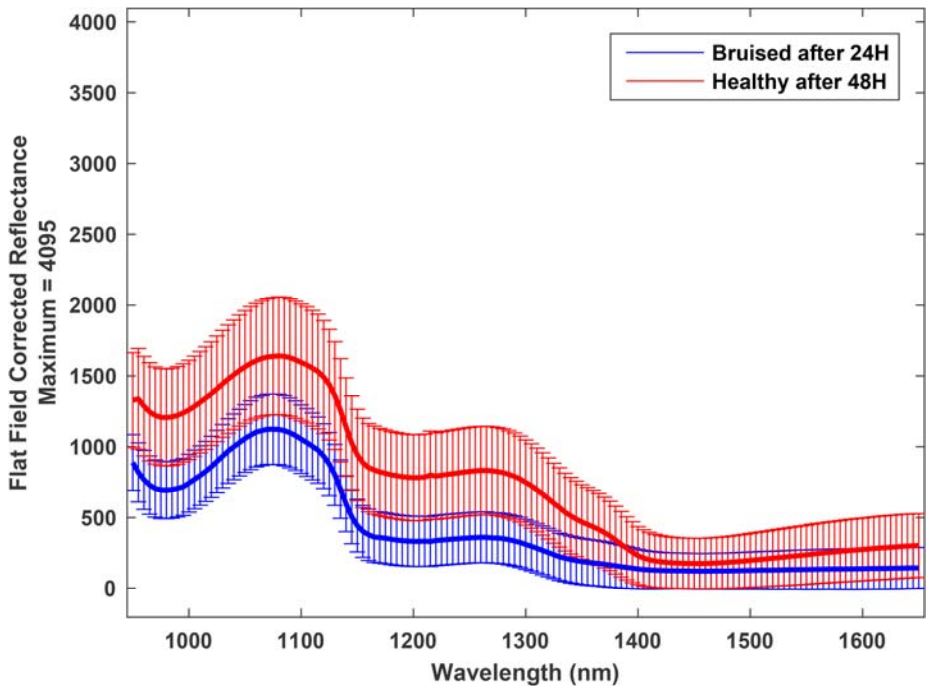

Figure S2. The spectra of healthy tissues stored for 48 hours and bruised tissues for 24 hours, respectively.

Table S1. MANOVA test criteria and exact F statistics for the hypothesis of no overall treatment effect between the spectra of healthy tissues stored for 48 hours and bruised tissues for 24 hours (H = Type III SSCP matrix for treatment, E = error SSCP matrix, S=1, M=69.5, N=8931, alpha=0.05).

Statistic	Value	F Value	Num DF	Den DF	p-value
Wilks' Lambda	0.14786372	730.14	141	17864	<.0001
Pillai's Trace	0.85213628	730.14	141	17864	<.0001
Hotelling-Lawley Trace	5.76298441	730.14	141	17864	<.0001
Roy's Greatest Root	5.76298441	730.14	141	17864	<.0001

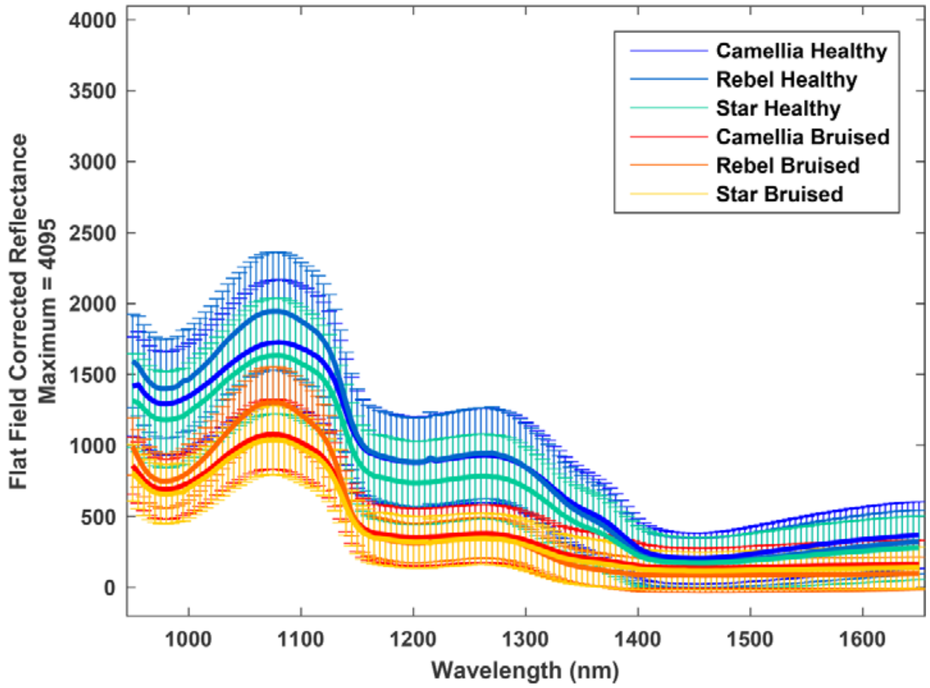

Figure S3. The spectra of healthy and bruised tissues for each of the southern highbush blueberry (SHB) cultivars.

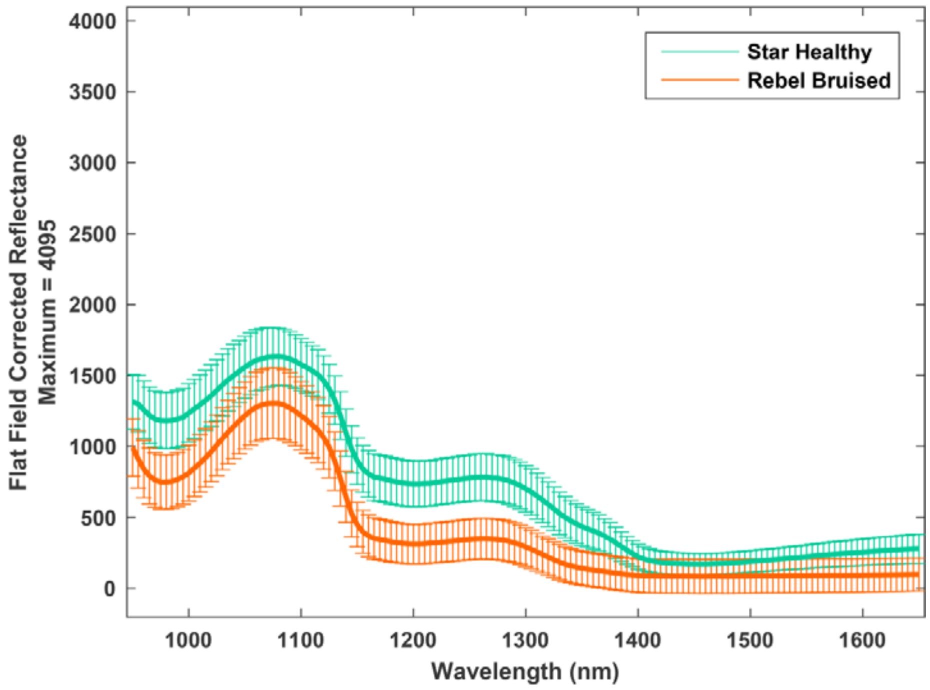

Figure S4. The closet spectra of healthy and bruised tissues for the southern highbush blueberry (SHB) cultivars.

Table S2. MANOVA test criteria and exact F statistics for the hypothesis of no overall treatment effect between the spectra of healthy tissues (Star) and bruised tissues (Rebel) (H = Type III SSCP matrix for treatment, E = error SSCP matrix, S=1, M=69.5, N=3396, alpha=0.05).

Statistic	Value	F Value	Num DF	Den DF	p-value
Wilks' Lambda	0.18422148	213.37	141	6794	<.0001
Pillai's Trace	0.81577852	213.37	141	6794	<.0001
Hotelling-Lawley Trace	4.42824853	213.37	141	6794	<.0001
Roy's Greatest Root	4.42824853	213.37	141	6794	<.0001

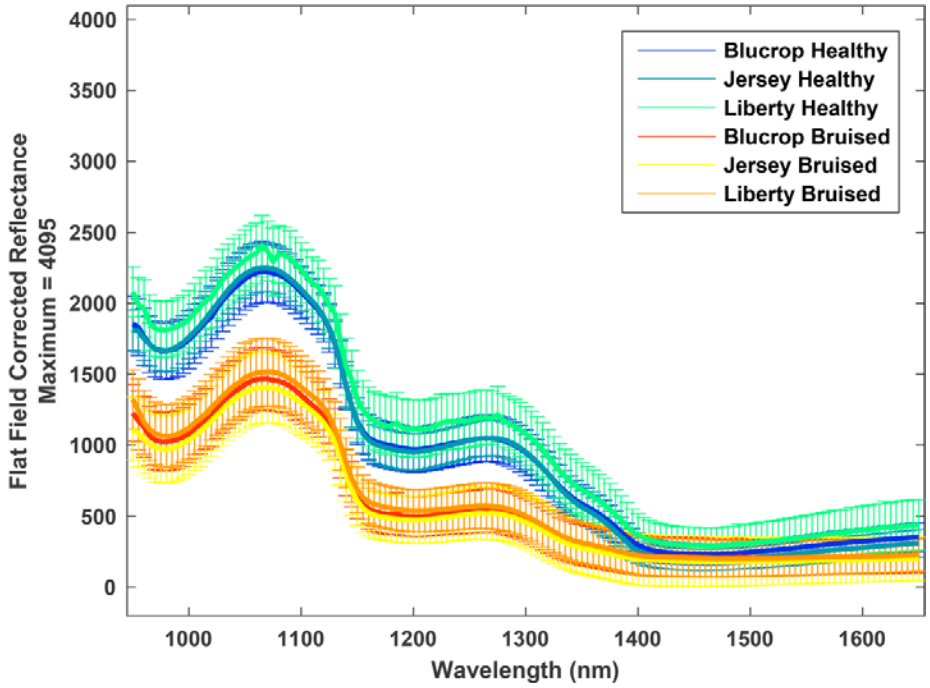

Figure S5. The spectra of healthy and bruised tissues for each of the northern highbush blueberry (NHB) cultivars.

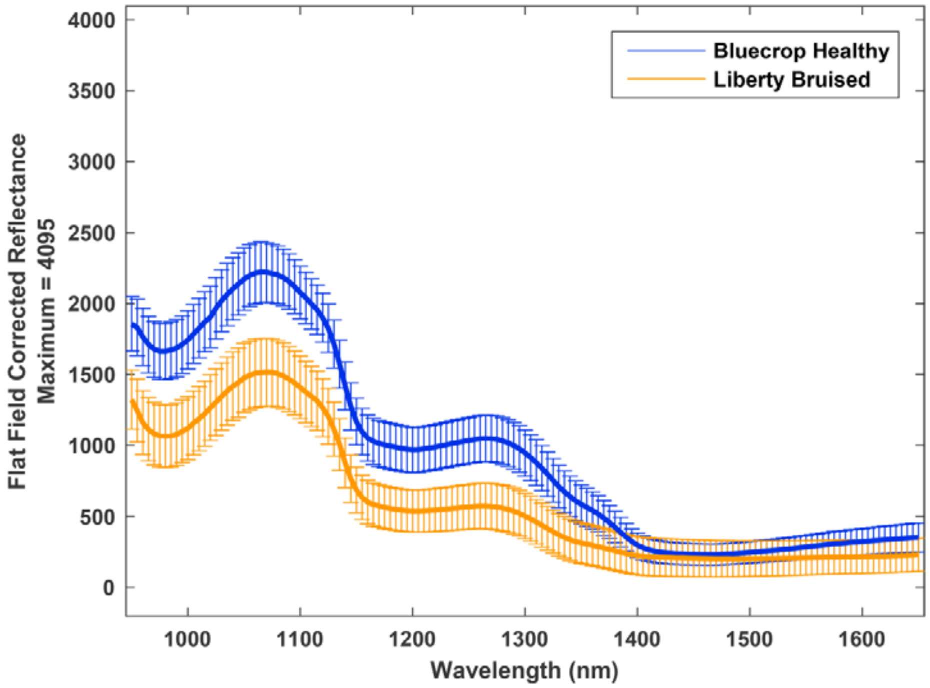

Figure S6. The closet spectra of healthy and bruised tissues for the northern highbush blueberry (NHB) cultivars.

Table S3. MANOVA test criteria and exact F statistics for the hypothesis of no overall treatment effect between the spectra of healthy tissues (Bluecrop) and bruised tissues (Liberty) (H = Type III SSCP matrix for treatment, E = error SSCP matrix, S=1, M=69.5, N= 35673.5, alpha=0.05).

Statistic	Value	F Value	Num DF	Den DF	p-value
Wilks' Lambda	0.26935836	1372.60	141	71349	<.0001
Pillai's Trace	0.73064164	1372.60	141	71349	<.0001
Hotelling-Lawley Trace	2.71252637	1372.60	141	71349	<.0001
Roy's Greatest Root	2.71252637	1372.60	141	71349	<.0001

Table S4. Multiple comparisons associated with Kruskal-Wallis test for 4 treatments in Figure 5 (a1) using measured firmness (H0: no statistical difference, alpha=0.05, $N_{control}$=30, N_{drop}=90).

	Diff	Lower	Upper	Decision	P-value
Control vs Drop height 15 cm	70.59444	22.34647	118.8424	Reject H0	0.00068
Control vs Drop height 23 cm	136.9056	88.65759	185.1535	Reject H0	0
Drop height 15 cm vs 23 cm	66.31111	32.19464	100.4276	Reject H0	2.00E-06
Control vs Drop height 31 cm	158.0556	109.8076	206.3035	Reject H0	0
Drop height 15 cm vs 31 cm	87.46111	53.34464	121.5776	Reject H0	0
Drop height 23 cm vs 31 cm	21.15	-12.9665	55.26647	FTR H0	0.611604

Table S5. Multiple comparisons associated with Kruskal-Wallis test for 4 treatments in Figure 5 (a2) using the bruise ratio index (H0: no statistical difference, alpha=0.05, $N_{control}$=30, N_{drop}=90).

	Diff	Lower	Upper	Decision	P-value
Control vs Drop height 15 cm	-55.8333	-104.081	-7.58536	Reject H0	0.013592
Control vs Drop height 23 cm	-113.111	-161.359	-64.8631	Reject H0	0
Drop height 15 cm vs 23 cm	-57.2778	-91.3943	-23.1613	Reject H0	5.70E-05
Control vs Drop height 31 cm	-124.611	-172.859	-76.3631	Reject H0	0
Drop height 15 cm vs 31 cm	-68.7778	-102.894	-34.6613	Reject H0	1.00E-06
Drop height 23 cm vs 31 cm	-11.5	-45.6165	22.61647	FTR H0	1

Table S6. Multiple comparisons associated with Kruskal-Wallis test for 5 treatments in Figure 5 (a4) using measured firmness (H0: no statistical difference, alpha=0.05, N=300).

	Diff	Lower	Upper	Decision	P-value
Control vs Fully-bruised	854.41	755.1333	953.6867	Reject H0	0
Control vs Drop height 60 cm	234.4733	135.1966	333.75	Reject H0	0
Fully-bruised vs Drop height 60 cm	-619.937	-719.213	-520.66	Reject H0	0
Control vs Drop height 120 cm	433.3967	334.12	532.6734	Reject H0	0
Fully-bruised vs Drop height 120 cm	-421.013	-520.29	-321.737	Reject H0	0
Drop height 60 cm vs 120 cm	198.9233	99.64663	298.2	Reject H0	0
Control vs Drop height 120 cm (Padded)	50.93667	-48.34	150.2134	FTR H0	1
Fully-bruised vs Drop height 120 cm (Padded)	-803.473	-902.75	-704.197	Reject H0	0
Drop height 60 cm vs 120 cm (Padded)	-183.537	-282.813	-84.26	Reject H0	2.00E-06
Drop height 120 cm vs 120 cm (Padded)	-382.46	-481.737	-283.183	Reject H0	0

Table S7. Multiple comparisons associated with Kruskal-Wallis test for 5 treatments in Figure 5 (a5) using the bruise ratio index (H0: no statistical difference, alpha=0.05, N=300).

	Diff	Lower	Upper	Decision	P-value
Control vs Fully-bruised	-867.857	-967.133	-768.58	Reject H0	0
Control vs Drop height 60 cm	-121.58	-220.857	-22.3033	Reject H0	0.005868
Fully-bruised vs Drop height 60 cm	746.2767	647	845.5534	Reject H0	0
Control vs Drop height 120 cm	-329.813	-429.09	-230.537	Reject H0	0
Fully-bruised vs Drop height 120 cm	538.0433	438.7666	637.32	Reject H0	0
Drop height 60 cm vs 120 cm	-208.233	-307.51	-108.957	Reject H0	0
Control vs Drop height 120 cm (Padded)	-54.05	-153.327	45.22671	FTR H0	1
Fully-bruised vs Drop height 120 cm (Padded)	813.8067	714.53	913.0834	Reject H0	0
Drop height 60 cm vs 120 cm (Padded)	67.53	-31.7467	166.8067	FTR H0	0.562104
Drop height 120 cm vs 120 cm (Padded)	275.7633	176.4866	375.04	Reject H0	0

Table S8. Multiple comparisons associated with Kruskal-Wallis test for 4 treatments in Figure 5 (a3) using the firmness predicted by PLSR (H0: no statistical difference, alpha=0.05, $N_{control}$=30, N_{drop}=90).

	Diff	Lower	Upper	Decision	P-value
Control vs Drop height 15 cm	90.05556	41.80854	138.3026	Reject H0	5.00E-06
Control vs Drop height 23 cm	116.7111	68.4641	164.9581	Reject H0	0
Drop height 15 cm vs 23 cm	26.65556	-7.46024	60.77135	FTR H0	0.235624
Control vs Drop height 31 cm	125.4556	77.20854	173.7026	Reject H0	0
Drop height 15 cm vs 31 cm	35.4	1.28421	69.51579	Reject H0	0.037137
Drop height 23 cm vs 31 cm	8.74444	-25.3714	42.86024	FTR H0	1

Table S9. Multiple comparisons associated with Kruskal-Wallis test for 5 treatments in Figure 5 (a6) using the firmness predicted by PLSR (H0: no statistical difference, alpha=0.05, N=300).

	Diff	Lower	Upper	Decision	P-value
Control vs Fully-bruised	785.1067	685.83	884.3834	Reject H0	0
Control vs Drop height 60 cm	46.92	-52.3567	146.1967	FTR H0	1
Fully-bruised vs Drop height 60 cm	-738.187	-837.463	-638.91	Reject H0	0
Control vs Drop height 120 cm	262.74	163.4633	362.0167	Reject H0	0
Fully-bruised vs Drop height 120 cm	-522.367	-621.643	-423.09	Reject H0	0
Drop height 60 cm vs 120 cm	215.82	116.5433	315.0967	Reject H0	0
Control vs Drop height 120 cm (Padded)	0.51667	-98.76	99.79337	FTR H0	1
Fully-bruised vs Drop height 120 cm (Padded)	-784.59	-883.867	-685.313	Reject H0	0
Drop height 60 cm vs 120 cm (Padded)	-46.4033	-145.68	52.87337	FTR H0	1
Drop height 120 cm vs 120 cm (Padded)	-262.223	-361.5	-162.947	Reject H0	0

Table S10. ANOVA with post hoc Tukey tests of the bruised fruit number calculated using the bruise ratio index and human assessment for various treatments using Bluecrop cultivar (alpha=0.05, N=4)

Treatment	Mean BFN (HA)	Mean BFN (BR)	Tukey group	p-value
Control	11	0	Different	0.0001
Fully-bruised	25	25	Same	n/a
Drop height 60 cm	10.5	1.5	Different	<0.0001
Drop height 120 cm (Steel)	13.5	8.5	Same	0.0839
Drop height 120 cm (Padded)	6	1	Different	0.0015

Note: BFN (bruised fruit number per treatment replicate), HA (human assessment), BR (bruise ratio index)

Table S11. ANOVA tests of the bruised fruit number calculated using the bruise ratio index and human assessment for various treatments using Jersey cultivar (alpha=0.05, N=4)

Treatment	Mean BFN (HA)	Mean BFN (BR)	Tukey group	p-value
Control	1.5	1.5	Same	1
Fully-bruised	25	25	Same	n/a
Drop height 60 cm	23.5	19.25	Same	0.0832
Drop height 120 cm (Steel)	13.5	8.5	Different	0.0074
Drop height 120 cm (Padded)	9.5	7	Same	0.6365

Note: BFN (bruised fruit number per treatment replicate), HA (human assessment), BR (bruise ratio index)

Table S12. ANOVA tests of the bruised fruit number calculated using the bruise ratio index and human assessment for various treatments using Liberty cultivar (alpha=0.05, N=4)

Treatment	Mean BFN (HA)	Mean BFN (BR)	Tukey group	p-value
Control	0.5	0	Same	0.1340
Fully-bruised	25	25	Same	n/a
Drop height 60 cm	2	0.25	Same	0.0584
Drop height 120 cm (Steel)	8	5.25	Same	0.0815
Drop height 120 cm (Padded)	2	0.25	Same	0.0584

Note: BFN (bruised fruit number per treatment replicate), HA (human assessment), BR (bruise ratio index)

Table S13. Summary of the two experiments conducted in this research

Variables	Experiment (Dataset) #1	Experiment (Dataset) #2
Cultivar	Camellia, Rebel, and Star (southern highbush cultivars)	Bluecrop, Jersey, and Liberty (northern highbush cultivars)
Total sample number	300 (100 per cultivar)	1500 (500 per cultivar)
Treatment	Control, three bruise treatments dropped from 15, 23, and 31 cm onto steel surface	Control, fully-bruised treatment (dropped from 90 cm onto steel surface for 8 times), three bruise treatments (60 and 120 cm onto steel and 120 cm onto padded surface
Treatment replicate	1	4
Sample number per treatment replicate	5 for control, 45 for bruise treatments	25
Bruising creation	Pendulum	Random
Bruising position	Stem, calyx, or equatorial axis	Random
Bruise development time	24 and 48 hours	24 hours

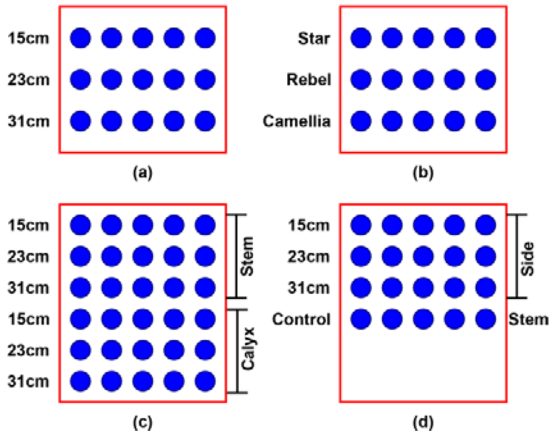

Figure S7. Layouts of hyperspectral images acquired for the southern highbush blueberry cultivars stored for 24 and 48 hours, respectively.

For the samples stored for 24 hours, 9 images contained 15 (5 sample replicates * 3 treatments) blueberry samples each from the same cultivar that had bruises at the same side, and each of the remaining 3 images contained 15 (5 sample replicates * 3 cultivars) blueberry samples of control treatment that the same side was positioned toward the camera. For the samples stored for 48 hours, each cultivar had four images. The first image contained 30 (5 sample replicates * 3 treatments * 2 hitting points) samples that had bruises at the stem and calyx end. The second image contained 15 (5 sample replicates * 3 treatments) samples that had bruises on the equatorial axis and 5 control group samples that its stem side was positioned toward the camera. The third and fourth images contained 5 control group samples that the calyx side and equatorial axis were positioned toward the camera, respectively. Table S1 includes the detailed layout for each hyperspectral images acquired for the southern highbush blueberry cultivars.

Table S14. Detailed treatment information and layout for the hyperspectral images acquired for the southern highbush blueberry cultivars.

Image	Cultivar	Treatment	Layout
HSI-1-1 to HSI-1-3	Camellia		Figure S5 (a)
HSI-1-4 to HSI-1-6	Rebel		Figure S5 (a)
HSI-1-7 to HSI-1-9	Star		Figure S5 (a)
HSI-1-10 to HSI-1-12		Control	Figure S5 (b)
HSI-2-1	Star		Figure S5 (c)
HSI-2-2	Star		Figure S5 (d)
HSI-2-3	Star	Control (calyx end)	
HSI-2-4	Star	Control (equatorial axis)	
HSI-2-5	Rebel		Figure S5 (c)
HSI-2-6	Rebel		Figure S5 (d)
HSI-2-7	Rebel	Control (calyx end)	
HSI-2-8	Rebel	Control (equatorial axis)	
HSI-2-9	Camellia		Figure S5 (c)
HSI-2-10	Camellia		Figure S5 (d)
HSI-2-11	Camellia	Control (calyx end)	
HSI-2-12	Camellia	Control (equatorial axis)	
